# Anatomical variants of the medioplantar oblique ligament and inferoplantar longitudinal ligament: an MRI study

**DOI:** 10.1007/s00276-021-02860-0

**Published:** 2021-11-20

**Authors:** Paweł Szaro, Khaldun Ghali Gataa, Bogdan Ciszek

**Affiliations:** 1 Department of Radiology, Institute of Clinical Sciences, Sahlgrenska Academy, University of Gothenburg, Göteborgsvägen 31, 431 80 Gothenburg, Sweden; 2 Department of Musculoskeletal Radiology, Sahlgrenska University Hospital, Gothenburg, Sweden; 3 Department of Descriptive and Clinical Anatomy, Medical University of Warsaw, Warsaw, Poland

**Keywords:** Ligament, Magnetic resonance imaging, Foot, Ankle, Spring ligament, Anatomy, Plantar ligament

## Abstract

**Purpose:**

The spring ligament complex (SL) is the chief static stabilizer of the medial longitudinal foot arch. The occurrence of normal anatomical variants may influence radiological diagnostics and surgical treatment. The aim of this study was to evaluate anatomical variants of the part of SL located inferior to the talar head (i-SL), medioplantar oblique ligament (MPO) and inferoplantar longitudinal ligament (IPL).

**Methods:**

We included 220 MRI examinations of the ankle performed on a 3.0 T engine. Only patients with a normal SL were included. Two musculoskeletal radiologists assessed the examinations and Cohen’s kappa was used to assess agreement. Differences between groups were assessed using the chi-squared test; *p* < 0.05 was considered as significant. The final decision was made by consensus.

**Results:**

Most commonly, i-SL was composed of the two ligaments IPL and MPO *n* = 167 (75.9%); in this group, bifid ligaments occurred in 19.2%, most commonly in the MPO. A branch to the os cuboideum was seen in *n* = 17 (10.2%). Three ligaments were seen in *n* = 52 (23.6%). In this group, bifid ligaments occurred in 13.5%; most commonly, the IPL was bifid and a branch to the os cuboideum was noted in *n* = 6 (11.5%). In one case, *n* = 1 (0.04%), we identified MPO, IPL and two accessory ligaments. No significant relationship was noted between the number of ligaments, the presence of bifid ligaments and side or gender (*p* > 0.05). Conclusion. More than two aligaments were seen in 24.1% of examined cases, the most common variant was the presence of MPO, IPL and one accessory ligament.

## Introduction

The plantar calcaneonavicular ligament or spring ligament (SL) consists of the superomedial ligament (SM), medioplantar oblique ligament (MPO) and inferoplantar longitudinal ligament (IPL); however, the nomenclature of SL is somewhat unclear [20] and Terminologia Anatomica does not mention SM, MPO or IPL [7]. Some authors divide SL into the superomedial ligament and the inferior calcaneonavicular ligament [3].

SM is the most robust component of SL orientated approximately in the sagittal plane. SM stretches between the sustentaculum tali and the dorsomedial surface of the navicular bone. Its superficial outline is related to the posterior tibial tendon, while the deep outline is covered by the cartilage and related to the talar head [1, 3]. The superior outline of the SM merges with the tibiospring ligament [8].

In the horizontal plane and inferior to the talar head, IPL and MPO are located. IPL and MPO originate on the coronoid fossa located on the calcaneus. Both IPL and MPO inserts on the navicular bone, IPL on the lateral and inferior surface, while MPO on the inferior and medial [14, 15].

SM is orientated in parallel to the talar head and interconnects with the deltoid ligament (DL), forming an anatomical and functional unit with load-bearing features [1–3, 18]. Both structures (SL and DL) can be visualized on magnetic resonance imaging (MRI) [10, 15, 16]. MPO and IPL have more tensile load functions than SM [3].

MPO and IPL are located inferior to the talar head, limiting the spring recess, which should not be taken as a tear of the MPO or IPL. The recess communicates with the talocalcaneonavicular joint [4, 15]. The fluid in the recess should not be taken as an effusion in the talocalcaneonavicular joint. IPL originates between the middle and anterior facets of the calcaneus on a structure called the “coronoid fossa” and inserts into the navicular beak. MPO originates as IPL and inserts into the navicular tuberosity [4, 15].

The medial longitudinal arch of the foot is supported mainly by the tibialis posterior, DL, SL, the long plantar ligament and the plantar fascia [4, 15]. The interaction of the above-mentioned structures means that the reconstruction of only one of them brings less benefits than the comprehensive reconstruction of the most important ones [15].

The tibiospring ligament is an important part of the deltoid ligament which is located in the anterior and superficial part of the deltoid ligament. SL mergers with the tibiospring ligament structure and together contribute to stabilizing of the medial part of the foot. The rupture of the SM occurs often at the connection with the tibiospring ligament [8].

SL tear may be seen in patients with flat foot. Surgical treatment of flat foot aims to correct the talus and os naviculare, thus rebuilding the medial longitudinal arch of the foot [17]. Progress in anatomical knowledge of the complexity of the SL may contribute to the development of treatment techniques.

To the best of our knowledge, studies of the anatomical variants of MPO and IPL have not been conducted on a large population by MRI. The aim of this study was to evaluate the anatomical variations of MPO and IPL.

## Materials and methods

### Design

This was a retrospective study of clinically indicated MRI examinations of the ankle performed between 2017 and 2020. Our study included 119 females and 91 males; the age range was 18–68 years, with a mean of 37.7 years. The right ankle was examined in 95 cases, and the left ankle was examined in 125 cases.

### Sample size calculation

We used *N* = 220 MRI ankle examinations. The sample size was calculated using Power a priori, effect size = 0.3, α = 0.05, power = 0.95.

### Inclusion criteria

The MRI ankle examinations were performed between 2018 and 2021 for non-traumatic indications. The imaging was performed on a 3.0 Tesla MRI machine (Philips©). Field of view included in the anterior extension at least the base of the metatarsal and in the posterior extension the tuber calcanei. The inclusion criteria included use of a dedicated ankle coil and the availability of at least the following sequences: proton density (PD) or T2-weighted sequences without fat saturation in the sagittal, axial, and coronal planes to assess ligament structure. The other sequences, such as T1-weighted turbo spin echo (T1-TSE), PD with fat suppression, or short-T1 inversion recovery (STIR), were used to detect pathology. PD-weighted turbo spin echo (TSE): TE (the echo time) 45 ms, TR (the repetition time) 2800–5000 ms. T2-weighted (TSE): TE 60 ms, TR 3000–5000 ms. T1-weighted: TE 11.5 ms, TR 700–750 ms. Voxel 0.45 × 0.53 × 3.0 mm, slide thickness 3 mm, field of view (FOV) 14 cm. No interslice gap was in our protocol. A dedicated ankle coil was used for MRI acquisition.

### Exclusion criteria

**Fig. 1:**
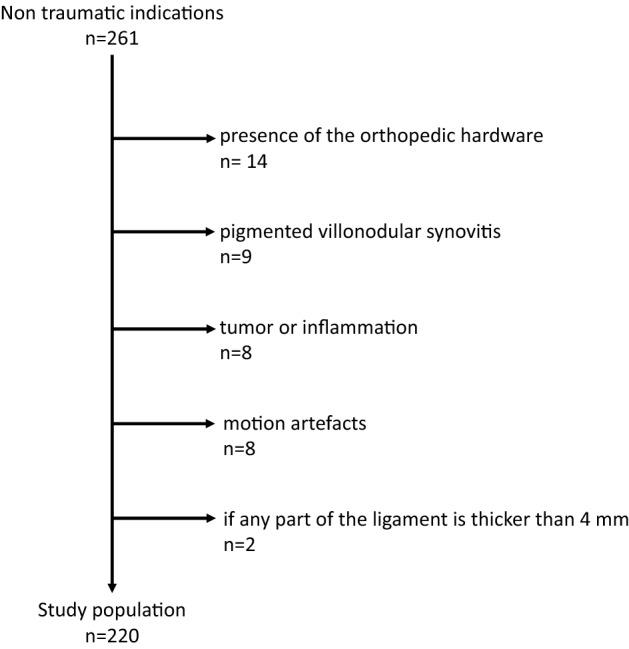
Study flow diagram

Exclusion criteria included abnormality of the superomedial part of the spring ligament complex, recent fracture, neoplasm, artifacts that might influence evaluation of the talus, navicular bone and calcaneus (e.g., metal artifacts or motion artefact), conditions that severely alter the appearance of the ankle (i.e., inflammation or tumor infiltration), and abnormality of the superomedial part of the spring ligament complex (SM). Abnormality of SM was considered abnormal if the ligament thickness was more than 4 mm, both with and without signal abnormality [13] (Fig. 1).

### Repeatability of the foot and ankle position

The patient was in supine position and the location of the ankle joint was maintained using a dedicated coil suited to the shape of the ankle and foot. Additional elastic wedge-shaped cushions were used to further secure the ankle and foot position. Thanks to this, it was not possible to change the position of the foot and ankle during the entire examination.

### Definitions and nomenclature

The most lateral ligament that runs laterally was called IPL, while the most medial which runs more medially was considered to be MPO. If the two separate fascicules could be distinguished at the navicular insertion, while the calcaneal insertion was common, a bifid ligament was considered. A separate ligament was considered if both the navicular and calcaneal insertion were separated, and two different structures were apparent.

If three separate ligaments were identified at the anatomical localization of MPO and IPL, a three fascicular variant was considered and the nomenclature was used as follows: the medial fascicle, the intermediate fascicle, and the lateral fascicle.

### Evaluation and study protocol

The MRI ankle evaluation was performed on a dedicated radiologic station separately by two musculoskeletal radiologists with 7 years (PS) and 5 years (KGG) of experience in radiology. A detailed clinical evaluation of the ankle was included in the referral letter. Uniform thickness, homogeneous signal, normal adjacent fat tissue, and no marrow edema or bone abnormalities indicate no trauma. The transverse plane was assessed parallel to the plantar surface of the foot.

IPL and MPO were found on horizontal sections based on the anatomical location. We then assessed where the IPL and MPO ended by analyzing subsequent cross-sections and whether there was a clear difference between the FR, which was considered as an absent connection. The presence of a connection was recognized when the structures smoothly merged into each other. Observations were confirmed on the other sections. The IPL and MPO were assessed if the separate or bifurcated fascicules were present. It was determined which of the fascicules was the widest in the horizontal plane. The presence of os trigonum and os calcaneus secundarius was assessed.

### Statistical analysis

Statistical analyses were carried out using the statistical package of SPSS 28.0 software for Apple (SPSS Inc., Chicago, USA). Cohen’s kappa was used to assess agreement between two raters. Values were interpreted as fair 0.21–0.40, moderate 0.41–0.60, substantial 0.61–0.80, and almost perfect 0.81–1.00 according to Landis et al. [12]. Differences between variables were assessed used the χ^2^ test. *p* < 0.05 was considered significant.

### Ethic approval

The Swedish Ethical Review Authority approved the study and waived the need for informed consent (number 2020-06177 and 2021-05447). Our study was performed in accordance with relevant named guidelines and regulations.

## Results

Anatomical variants of the plantar calcaneonavicular ligament are common and can be visualized on MRI. The highest agreement was noticed in the group of two independent IPL and MPO, the lowest agreement was noticed in group of the bifid IPL (Table 1). All Cohen’s kappa values were in the 95% CI (Table 1).

**Table 1:** Cohen’s kappa, bias, standard error, and confidence interval for the agreement in the inferoplantar longitudinal ligament and medioplantar oblique ligament evaluation

	Cohen’s kappa*	Bias	Std. error	95% confidence interval	
				Lower	Upper
Two fascicles (group A)	0.59	0.00	0.09	0.41	0.76
MPO larger than IPL	0.39	0.01	0.08	0.24	0.55
IPL larger than MPO	0.25	-0.01	0.10	-0.14	0.34
IPL and MPO of similar size	0.39	-0.02	0.11	0.16	0.62
MPO is bifid	0.38	0.01	0.11	0.17	0.59
IPL is bifid	0.52	-0.04	0.21	-0.02	0.80
Three fascicles (group B)	0.32	-0.01	0.09	0.12	0.49
The lateral fascicle is the biggest	0.37	0.00	0.12	0.11	0.56
The intermediate fascicle is the biggest	0.43	0.03	0.17	0.11	0.76
The medial fascicle is the biggest	0.32	0.01	0.09	0.13	0.66
The separate fascicle to os cuboideum	0.38	0.01	0.12	0.13	0.65
The navicular bone type 2	0.48	0.01	0.17	0.14	0.82
The navicular bone type 3	0.85	0.00	0.11	0.63	1.00

^*^P < 0.001

**Fig. 2:**
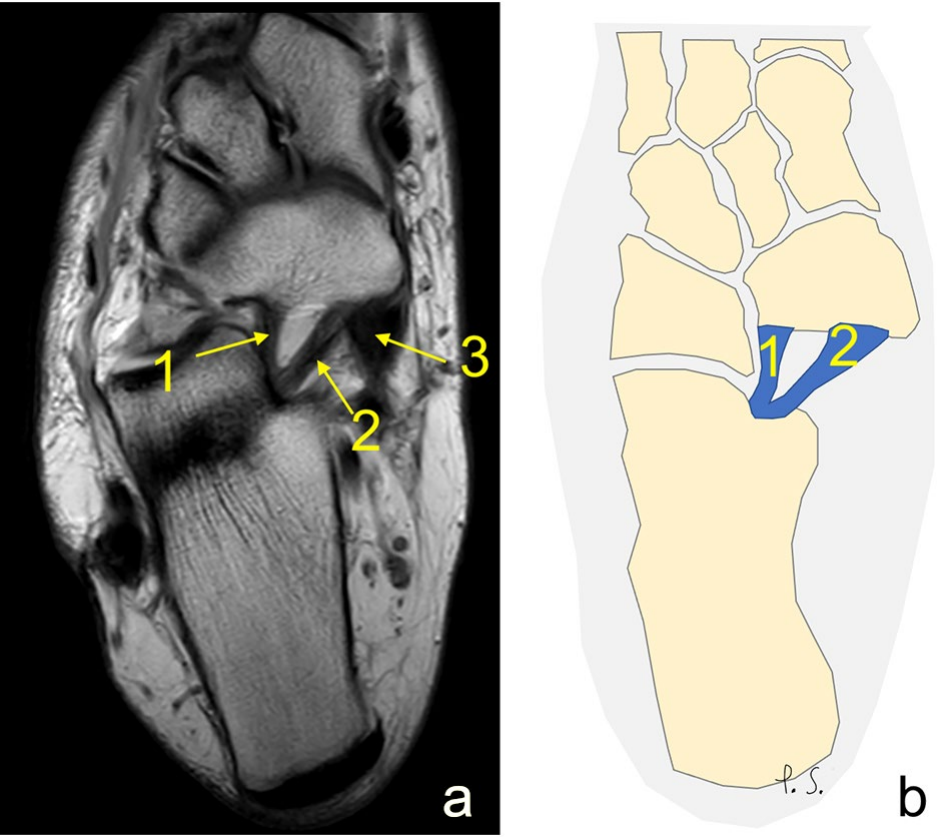
Two separate ligaments of similar size. **a** Proton density, axial section, **b** schematic diagram. 1—the inferoplantar longitudinal ligament, 2—the medioplantar oblique ligament, 3—the tibialis posterior tendon

**Fig. 3:**
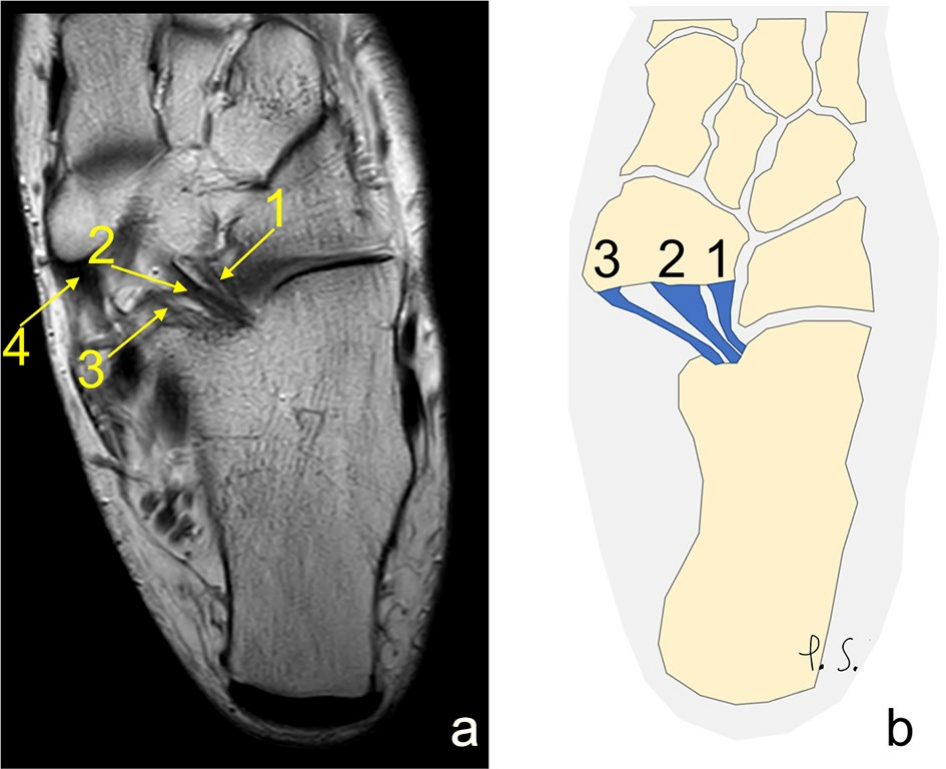
Three separate ligaments of similar dimensions. **a** proton density, axial section, **b** schematic diagram. 1—the lateral fascicle, 2—the intermediate fascicle, 3—the medial fascicle, 4—the tibialis posterior tendon

**Fig. 4:**
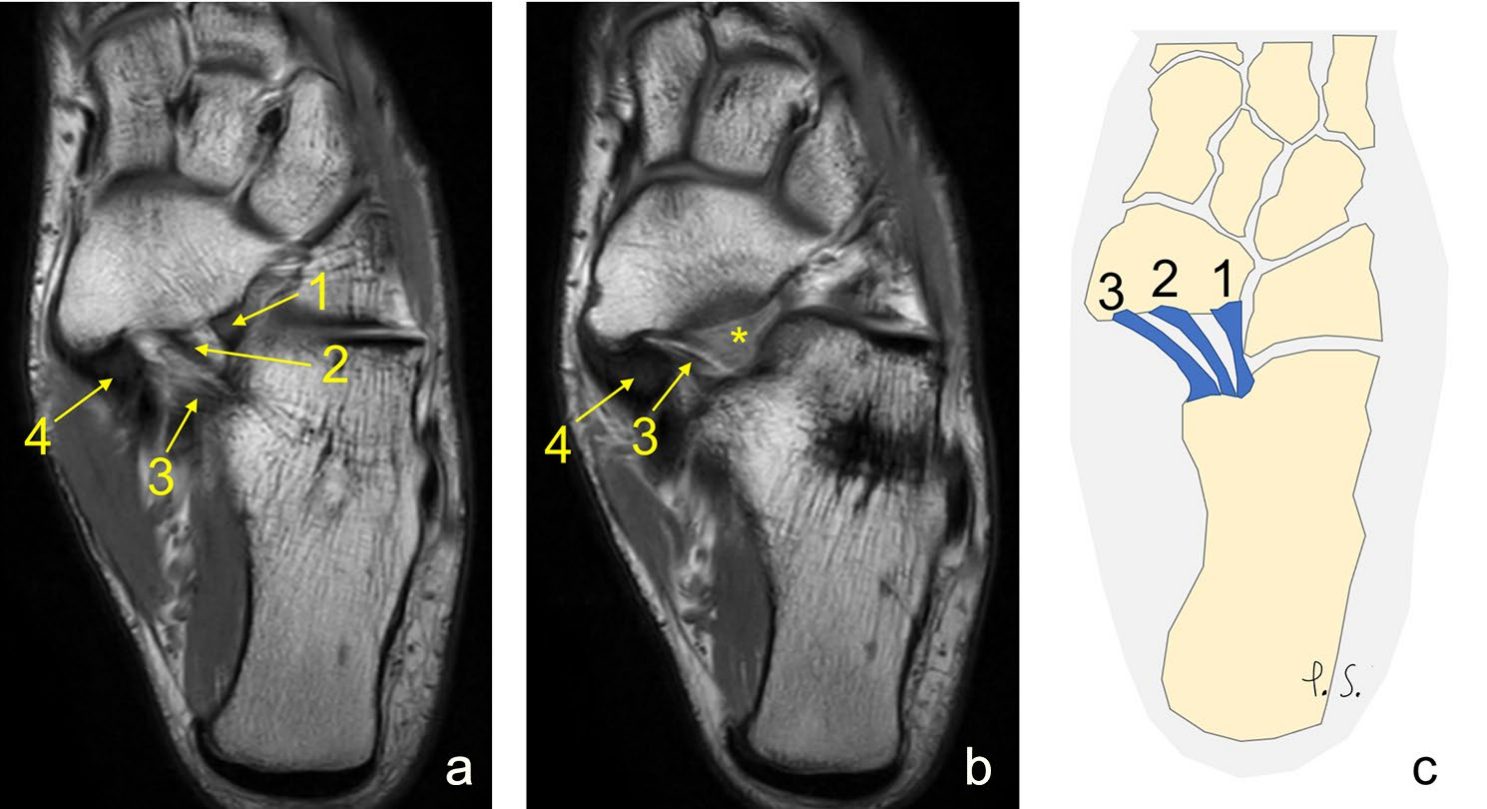
Three separate ligaments of different dimensions. **a**, **b** proton density, axial sections, **c** schematic diagram. 1—the lateral fascicle, 2—the intermediate fascicle, 3—the medial fascicle, 4—the posterior tibialis tendon, *—the partial volume effect of 1 and 2

**Fig. 5:**
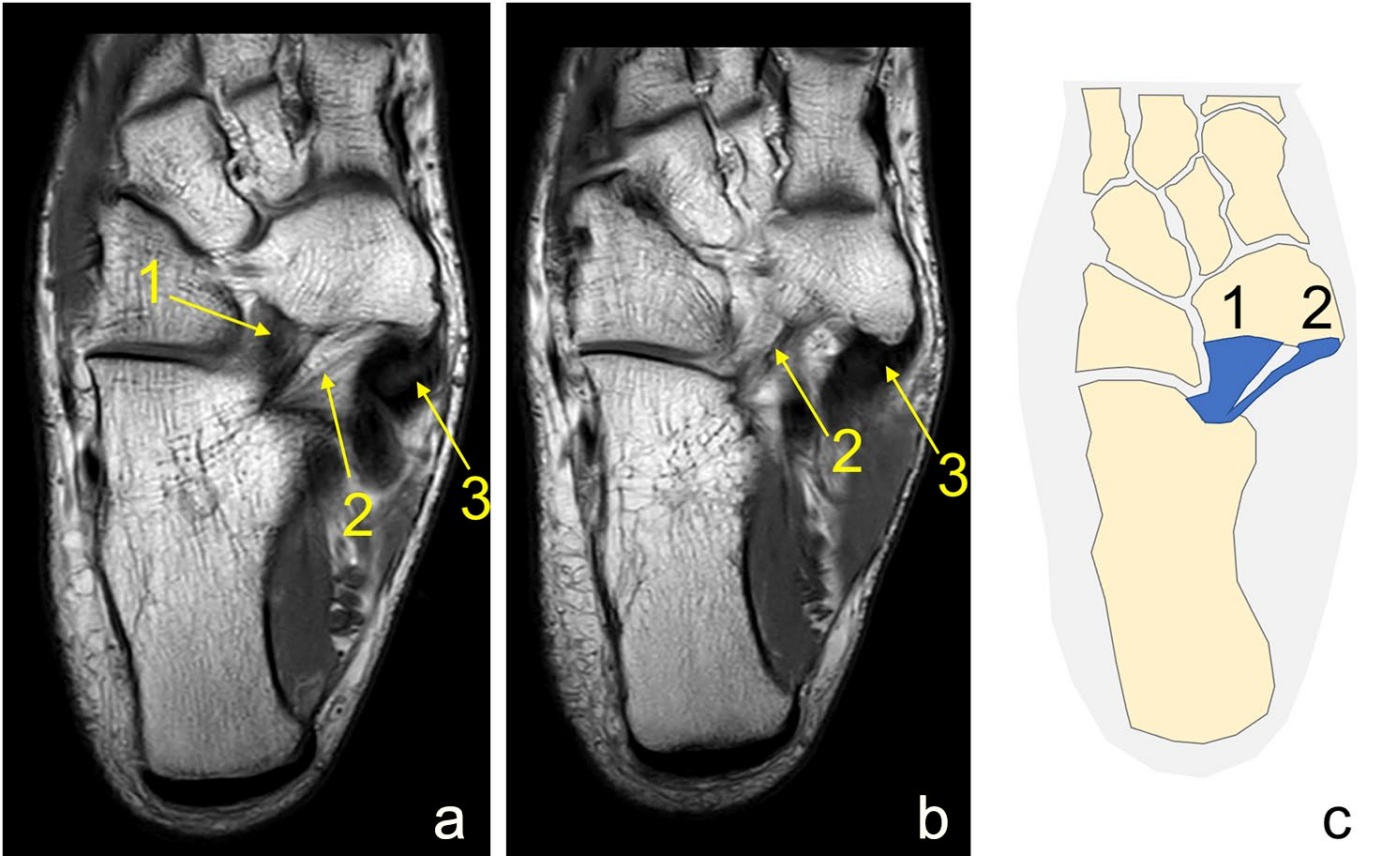
The inferoplantar longitudinal ligament is larger than the medioplantar oblique ligament. **a**, **b** proton density, axial sections, **c** schematic diagram. 1—the inferoplantar longitudinal ligament, 2—the medioplantar oblique ligament, 3—the tibialis posterior tendon

**Fig. 6:**
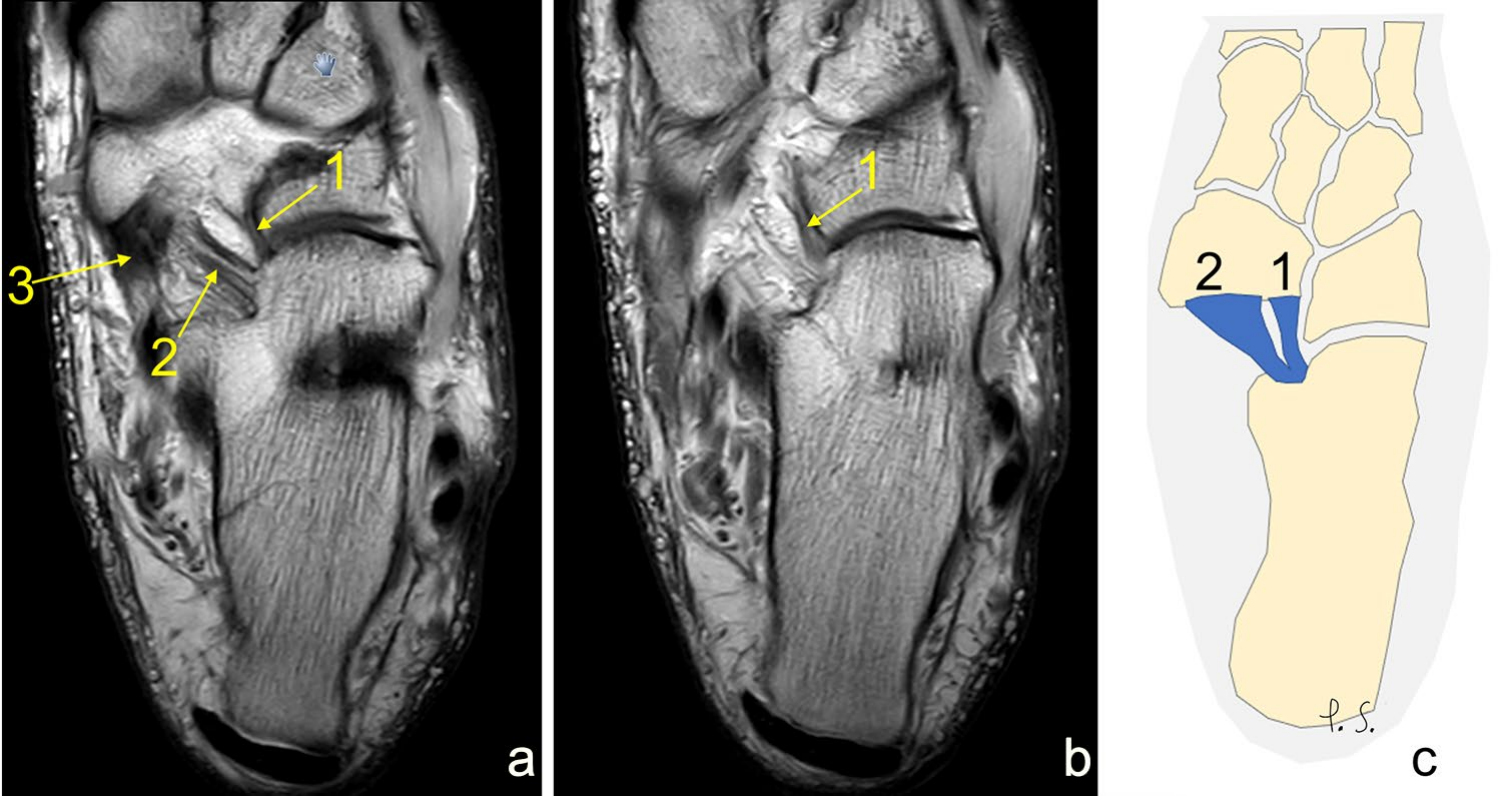
The medioplantar oblique ligament is larger than the inferoplantar longitudinal ligament. **a**, **b** proton density, axial sections, **c** schematic diagram. 1—the inferoplantar longitudinal ligament, 2—the medioplantar oblique ligament, 3—the tibialis posterior tendon

**Fig. 7:**
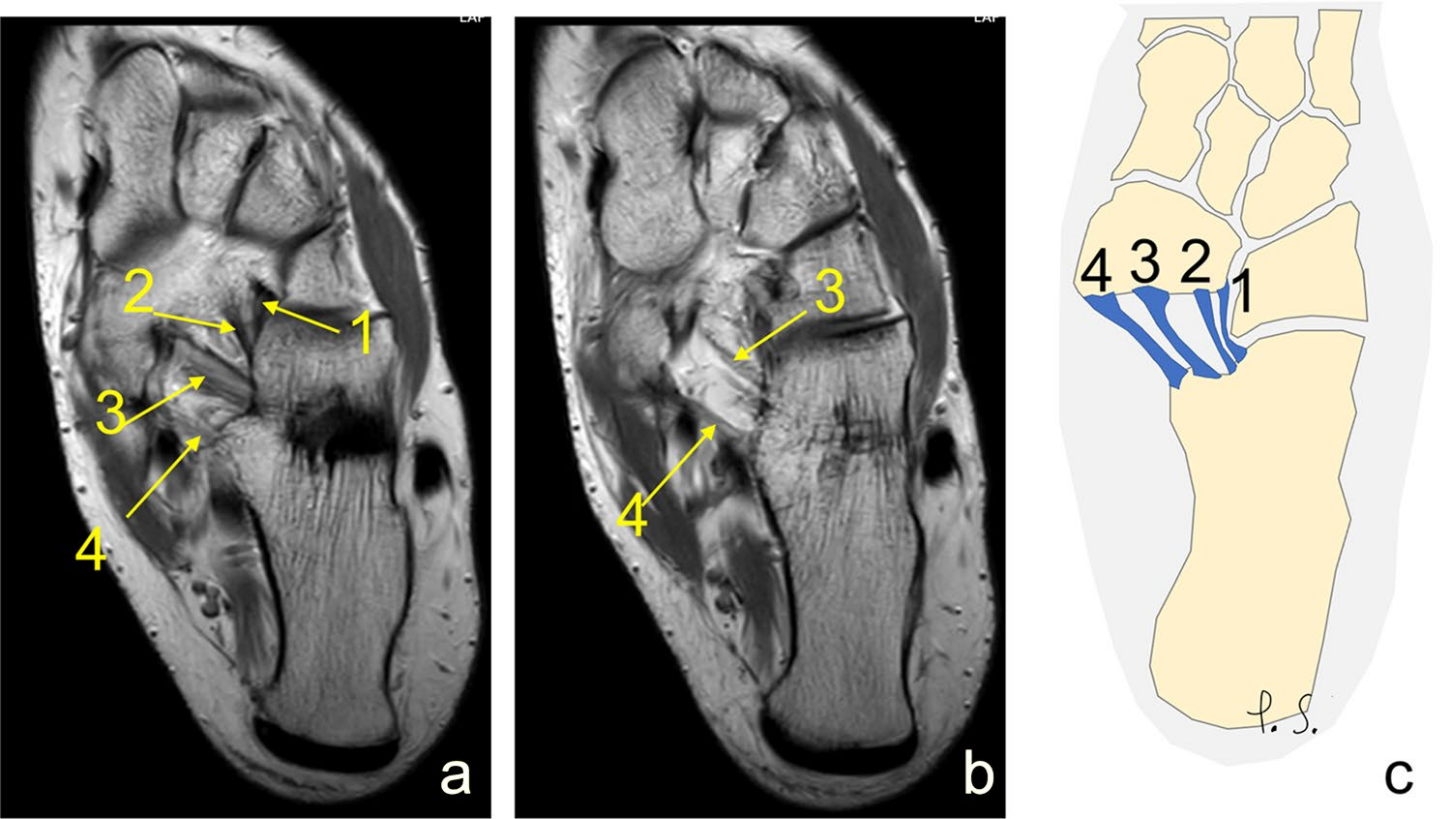
The four fascicular variant of the inferoplantar longitudinal ligament and the medioplantar oblique ligament. A and b- proton density, axial sections, c- schematic diagram. 1 to 4 show the four separate ligaments

In all cases (*N* = 220), MPO and IPL were identified. Most commonly, SL was composed of the two ligaments IPL and MPO *n* = 167 (75.9%); group A (Fig. 2). Three ligaments were seen in *n* = 52 (23.6%); group B, (Figs. 2, 3, 4). If two ligaments were present, the IPL was wider than MPO in 38.6% in the transverse section (Table 2, Figs. 5, 6). In one case, four ligaments were noted; group C, Fig. 7.

**Table 2:** Comparing two and three distinct ligaments

		IPL is the greatest ligament	MPO is the greatest ligament	MPO and IPL of similar size
Two distinct ligaments (group A) *n* = 167 (75.9%)	*n*	85	46	36
	%	38.6%	20.9%	16.4%

		The lateral fascicle is the greatest ligament	The medial fascicle is the greatest ligament	The intermediate fascicle is the greatest ligament
Three distinct ligaments (group B) *n* = 52 (23.6%)	n	31	12	9
	%	14.1%	5.5%	4.1%

**Fig. 8:**
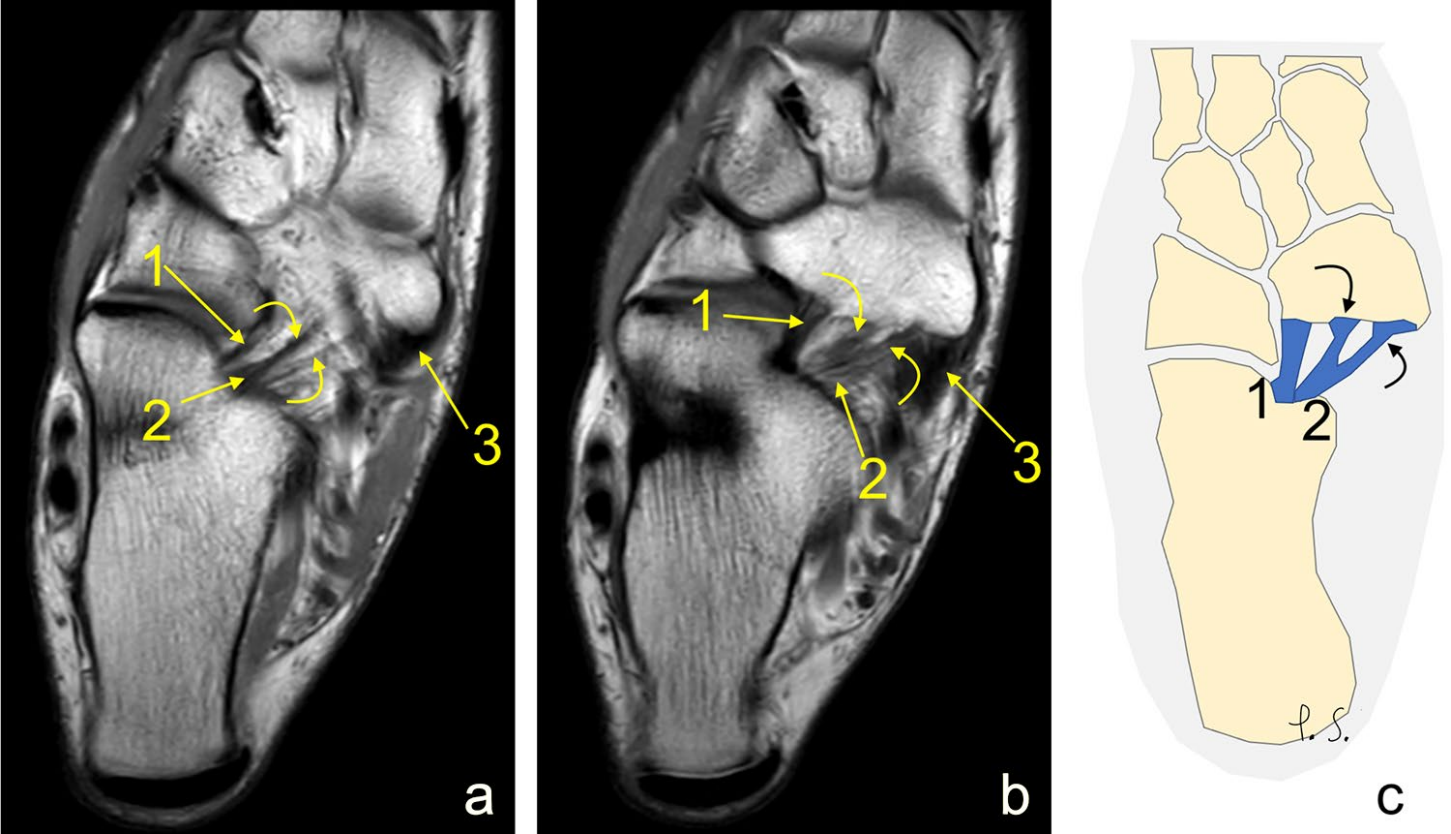
The medioplantar oblique ligament is bifid. **a**, **b** proton density, axial sections, **c** schematic diagram. 1—the inferoplantar longitudinal ligament, 2—the medioplantar oblique ligament, 3—the tibialis posterior tendon. Curved arrows showed two parts of the bifid medioplantar oblique ligament

**Fig. 9:**
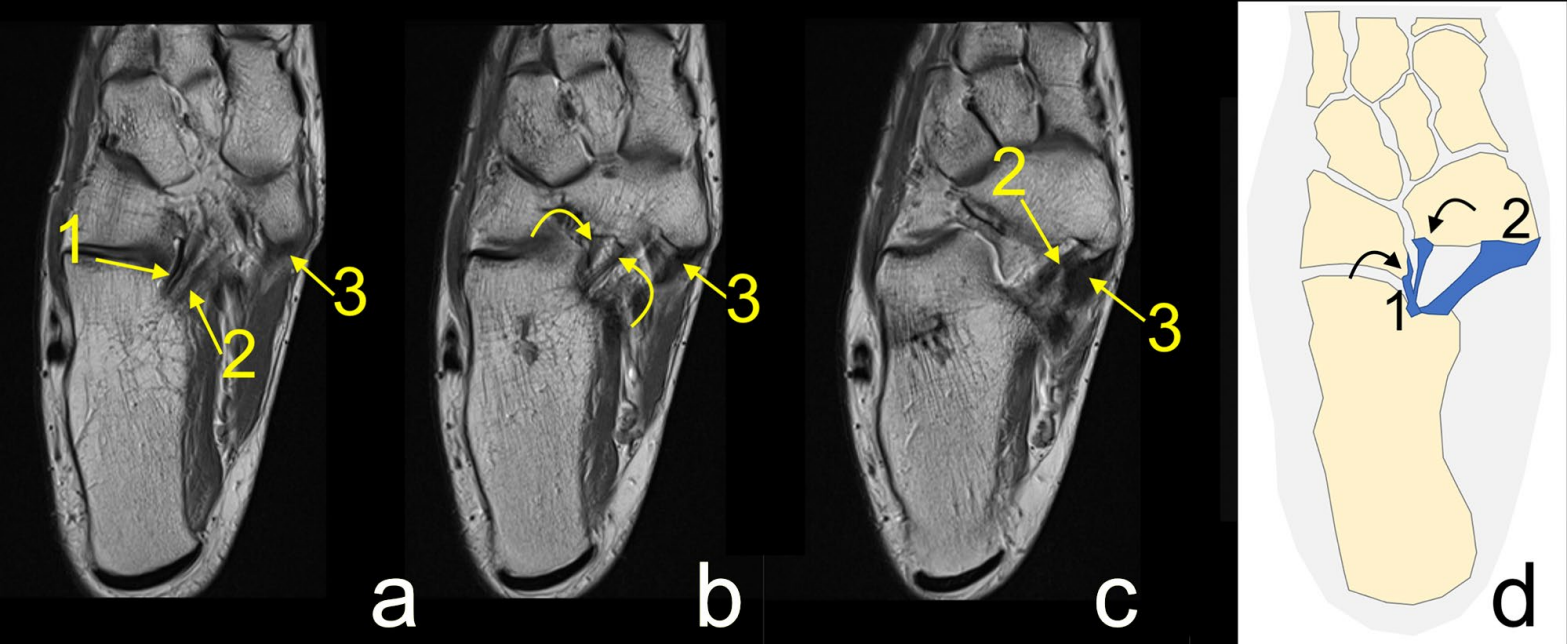
The inferoplantar longitudinal ligament is bifid. **a**–**c** proton density, axial sections, **d** schematic diagram. 1—the inferoplantar longitudinal ligament, 2—the medioplantar oblique ligament, 3—the tibialis posterior tendon. Curved arrows showed two parts of the bifid inferoplantar longitudinal ligament

In group A, bifid MPO was more common than bifid IPL (Table 3, Figs. 8, 9). In the group with bifid MPO and IPL, IPL was wider than MPO on the transverse plane. In group A, in 10.2%, the accessory fascicle to the cuboid bone was noted (Tables 4 and 5). In this subgroup A1, IPL was most commonly wider than MPO or bifid (Table 4). Bifid ligament variants were more commonly seen in the group with two ligaments (group A), *p* < 0.05 (Table 5, 6).

**Table 3:** Bifid ligaments in the group with two ligaments (group A)

	MPO is the greatest ligament	IPL is the greatest ligament	MPO and IPL of similar size
MPO bifid (*n* = 21)			
	5	10	6
	2.3%	4.5%	2.7%
IPL bifid (*n* = 11)			
	2	6	3
	0.9%	2.7%	1.4%

Bifid components at the navicular insertion

**Table 4:** Branch to the cuboid bone in group of two ligaments (group A)

MPO is the greatest ligament	IPL is the greatest ligament	MPO and IPL of similar size	MPO bifid	IPL bifid
3	10	4	2	9
1.4%	4.5%	1.8%	0.9%	4.1%

The branch to the cuboid bone was seen in *n* = 17 of group A

**Table 5:** Branch to the cuboid bone in the group with three ligaments (group B)

The medial fascicle is the greatest	The lateral fascicle is the greatest	The intermediate fascicle is the greatest	The medial fascicle is bifid	The lateral fascicle is bifid	The intermediate fascicle is bifid
3	3	0	0	3	0
1.4%	1.4%	0.0%	0.0%	1.4%	0.0%

The branch to the cuboid bone was seen in *n* = 6 in group B

**Table 6:** Bifid ligaments in the group with three ligaments (group B)

The lateral fascicle is bifid	The intermediate fascicle is bifid	The medial fascicle is bifid
5	0	2
2.3%	0.0%	0.9%

## Discussion

The most important finding of our study is that anatomical variants of the MPO and IPL are common. In about one quarter of cases, more than MPO and IPL were seen. The presence of a branch to the cuboid bone was noted nearly twice as often in the group with two ligaments compared to the group with three ligaments. Bifid ligaments were more much common in the group with two ligaments compared to the group with three ligaments. Agreement in the assessment in group of the two-fascicular variants was nearly twice as high as in the case of three-fascicular variants.

MPO and IPL comprise the spring ligament complex, together with SM. SL is a key static foot stabilizer that together with the tibialis posterior, which is a dynamic stabilizer, supports the medial foot arch [11]. Surgical reconstruction of the tibialis posterior and SL is required in acquired flat foot to obtain a good clinical outcome when both mentioned structures are injured [11]. Precise estimation of the degree of damage of the SL and tibialis posterior is essential before decision-making regarding the type of surgical reconstruction. The relatively frequent occurrence of anatomical variants of MPO and IPL may significantly influence the radiological evaluation. The presence of accessory fascicles, as well as bifid variants of MPO or IPL limiting the recess, may mimic a ligament tear. This may be particularly important because, in the case of anatomical variants, the agreement in our study was lower in the group with three fascicular variants (Group B) than in the case of the most common variants of MPO and IPL (Group A). MRI is the method of choice in the radiologic evaluation of SL and is often used before a preoperative evaluation [9, 16]. Anatomical variations of SL should be kept in mind in decision-making before surgery.

In the most common variation asymmetry of the MPO and IPL is visible. Anatomy is usually related to function. It is difficult to explain the origin and significance of asymmetry between MPO and IPL. However, we believe that there is a tendency to have smaller anatomical units in the ankle ligaments, which has been studied before [6, 19]. In our group, the presence of additional fascicles or bifurcated bundles was observed more often in the group with two ligaments (group A) than in the group with three ligaments (group B). SL with DL and the posterior tibial stabilize the medial part of the foot. Foot stabilization is based on several anatomical structures; however, their anatomical variants are relatively poorly understood. It cannot be ruled out that the relatively frequent anatomical variations of MPO and IPL may also be associated with variations of other structures responsible for the stabilization of the medial part of the foot.

MPO and IPL are separated by a thin layer of fat tissue from the tibialis posterior tendon [5]. The direct anatomical and functional relationship of SM with DL is known; thus, disorders of DL may influence SL and may cause collapse of the medial longitudinal arch of the foot. Similarly, dysfunction of the tibialis posterior tendon may stretch SM and cause inferior dislocation of the talar head, resulting in a reduction in the medial longitudinal arch, weaker push-off and finally valgus of the hindfoot [4]. Anatomical communications between the tibialis posterior tendon, DL, and SM are well-known [2, 3]. Unfortunately, the connection to the MPO or IPL has not been examined, to our knowledge. The presence of a communication between SM and MPO and IPL is somewhat unclear. Some authors state that it is not possible to distinguish between them, whereas others think the opposite [3, 20]. Quoted differences between old studies and more modern work probably depends on a more detailed methodology, as used currently. In our study, in all patients, we could identify MPO and IPL. Complexity and anatomical variations of SL may influence identification and problems with nomenclature. In some previous studies, a third component of SL, which was then renamed MPO, was found in direct relation to SM, which was covered by fibrocartilage [20]. There were some nomenclature discrepancies early on. Some authors classified this ligament as part of SL, while others consider is as a separate part from SM, because is separated by a fat plane [4, 20]. In our study, MPO could be always distinguished from the IPL. We did not find a single-fascicular variant in any case. In the examined material, we noticed a tendency to the appearance of several fascicles, and in the case of two-fascicular variants, we observed a tendency to division or the presence of additional branches to the os cuboideum. A similar observation was noted in a previous MRI study where the presence of a fluid-filled space separating IPL and MPO was noted in all examined cases, similar to our study [4]. Previous studies showed differences between the components of SL regarding the orientation of the fibers, histological differences and chemical composition [9], which may confirm our hypothesis that smaller parts are probably responsible for stabilization in different positions of the foot.

MPO and IPL were found in all cases, in contrast to some previous studies [14]. The difference may be consequential in terms of population differences and different MRI protocols. In most cases, MPO and IPL were noted; however, differences in size and the presence of bifid ligaments were observed, which corresponds to the tendency to divide into smaller parts.

The presence of anatomical variants shows that the agreement of the MR assessment is lower than in typical cases. This may indicate a known problem in assessing rarer anatomical variants. The authors see the need for increased education regarding the presence of anatomical variants. The assessment of common anatomical variants such as os naviculare accesorium was associated with a higher agreement between observers than in the presence of additional fascicles or branches of MPO or IPL.

We acknowledge several limitations in the present study. Some factors may influence on MR appearance of MPO and IPL as chosen protocol, slide orientation, and partial volume effect. There are some physiological factors as mechanical adaptation, which may affect fascicular diameters. To minimize the impact of these factors, two radiologists independently evaluated each MRI examination, and the final result was done by consensus. Each examination was performed in a dedicated coil that ensured repeatable positioning of the foot. The cross-sections were always made in the same way, parallel to the plantar side of the foot. Ligament pathologies were ruled out using exclusion criteria.

There are several possible clinical applications of our results. Relatively frequent occurrence of anatomical variants of IPL and MPO should be considered when assessing MRI examinations and when making decisions about the choice of treatment. The results may be applied in the development of new techniques for ligament reconstruction. The presence of anatomical variations is also essential for understanding the biomechanics of the foot.

## Conclusions

Anatomical variants of MPO and IPL are common and can be visualized on MRI. The presence of anatomical variants decreases agreement between investigators, making MRI evaluation more difficult. Most commonly, the presence of an accessory ligament was noted. Bifurcated ligaments and an accessory branch to the cuboid bone were observed more often in the group with MPO and IPL compared to the group of three-ligament variants.

## Data Availability

Yes.

## Abbreviations

FOV: Field of view
i-SL: The part of the spring ligament which is located inferior to talar head
IPL: The inferoplantar longitudinal ligament
MPO: The medioplantar oblique ligament
PD: Proton density
SL: The spring ligament (inferior calcaneonavicular ligament)
SM: The superomedial ligament
TE: Echo time
TR: Repetition time
TSE: Turbo spin echo

## Code availability

Yes.
